# A response to Nasrallah et al. “Infection rates of trans-perineal versus trans-rectal prostate biopsy: A Middle Eastern tertiary center experience—Time for a change?”

**DOI:** 10.1080/20905998.2025.2585749

**Published:** 2025-11-13

**Authors:** Minesh Patel, Joseph Norris

**Affiliations:** Urology Department, Imperial College Healthcare NHS Trust, London, UK

Dear Professor,

We read with great interest the article by Nasrallah et al., *‘Infection rates of trans-perineal versus trans-rectal prostate biopsy: A Middle Eastern tertiary center experience – Time for a change?’*. The authors should be commended for addressing the increasingly important issue concerning infection risks in prostate biopsies and presenting their data from a tertiary centre in the Middle Eastern region. The data from this work supports the growing body of evidence favouring the transperineal (TP) approach over the transrectal (TRUS) route, demonstrating fewer urinary tract infections (1.2% vs 4.1%, *p* = 0.03) and lower hospitalisation rates.

However, several aspects of the study merit further consideration. Firstly, is the heterogeneity in antibiotic prophylaxis, ranging from fluoroquinolones to aminoglycosides and cephalosporins. This introduces a significant degree of confounding and without stratified subgroup analysis or adjustment for antibiotic regime, it is difficult to attribute infection rate differences solely to biopsy approach. Additionally, the study reports that several operators transitioned from TRUS to TP during the study period. Even for experienced urologists, the transperineal approach requires adaptation to new access routes, needle trajectories and anaesthetic methods. This introduces a learning curve in which infection rates in the TP cohort may have been higher in the initial phase following this transition. Further to this, it is worth exploring whether there was a selection bias in which potentially higher risk patients were preferentially assigned to one technique. Nevertheless, both aspects could have influenced the outcomes recorded.

Furthermore, it is worth noting that previous trials such as PROBE-PC and PREVENT have found no discernible difference between the techniques in terms of infection-related complications [1,2] (PROBE-PC: *n* = 718, 3% [10/367] LATP *vs* 3% [9/351] TRUS; PREVENT: *n* = 567, 0% [0/287] LATP *vs* 1% [4/280] TRUS, *p* = 0·059). Fewer infections in the TP group were recorded in the TRANSLATE trial however this was not statistically significant and as infection-related complications were a secondary outcome, the study was not powered on this [3]. In this context, other aspects of the debate between the TRUS and TP biopsy techniques are worth exploring. This includes comparative data on diagnostic yield and accuracy, sampling adequacy which would add more value and context. In particular, the TRANSLATE trial has demonstrated a greater detection of Gleason grade group 2 (GGG2) or higher disease with TP biopsies in comparison to TRUS in biopsy-naïve patients [3].

Despite these limitation, Nasrallah et al have contributed valuable regional evidence that supports the global transition between TP biopsy as the safer standard of care. This work highlights the need for further multicentre studies with standardised antibiotic and procedural protocols to better our understanding of infection risk and to develop universal best practice for prostate biopsy.

## Response to the Letter to the Editor

We would like to sincerely thank Dr. Minesh Patel and Dr. Joseph Norris for their insightful comments regarding our article, “Infection Rates of Trans-perineal versus Trans-rectal Prostate Biopsy: A Middle Eastern Tertiary Center Experience—Time for a Change?” We appreciate their interest and the opportunity to clarify several important points raised in their correspondence. We will be addressing the points raised by order mentioned in the response letter.

**1. Antibiotic Prophylaxis**

The heterogeneity in antibiotic prophylaxis indeed reflects the evolving practice pattern at our institution during the transition from transrectal (TRUS) to transperineal (TP) prostate biopsy. In our setting, both fluoroquinolones and cephalosporins were used interchangeably and were considered within the same prophylactic category. Aminoglycosides, on the other hand, were frequently added, particularly in patients with a history of urinary tract infections or prior infections caused by quinolone or cephalosporin resistant organisms. Therefore, antibiotic prophylaxis in our cohort was not a determinant of infection risk between the TP and TRUS groups, but rather a reflection of individualized management within the TRUS group itself, where higher-risk patients often received combination coverage. Importantly, TP biopsy patients did not routinely receive antibiotic prophylaxis, particularly in the last two years of the study period, as institutional policy evolved toward omitting antibiotics altogether for TP procedures. This change was based on both local and international data demonstrating the extremely low infectious risk associated with the TP route. As such, the difference in infection rates observed between TP and TRUS biopsy in our study is less likely to be confounded by antibiotic variability.

**2. Operator Transition and Learning Curve**

We acknowledge the potential impact of operator transition and the learning curve. However, given the high procedural volume at our institution, and the extensive prior experience of our attending urologists (each with over 500 prostate biopsies performed), the learning curve was short and steep, with minimal influence on procedural outcomes. While the learning curve is an inherent factor in any evolving surgical practice, we believe its effect in our series was minor but possibly may have altered the observed difference in infection rates.

**3. Selection Bias**

The selection of biopsy approach (TRUS vs TP) was determined solely by individual attending preference rather than patient characteristics. Two of our attending urologists have continued to perform transrectal biopsies exclusively, whereas the remaining attendings have adopted the transperineal approach as their standard of care. Consequently, patients were not assigned to either technique based on risk profile, comorbidities, or clinical presentation, minimizing the risk of selection bias within our dataset.

**4. Comparison with Randomized Controlled Trials**

We agree that randomized controlled trials such as PROBE-PC, PREVENT, and TRANSLATE provide valuable evidence for comparing TRUS and TP techniques. However, our study differs in design and context. As a retrospective single-center analysis, it inherently carries limitations related to data completeness and potential underreporting of mild infections not captured in our institutional database. Nonetheless, our findings represent real-world evidence from a Middle Eastern tertiary center with distinct antimicrobial resistance patterns, providing valuable insight into the safety and feasibility of the TP approach in regions with high baseline infection rates and quinolone resistance.

We once again thank Dr. Patel and Dr. Norris for their thoughtful critique and acknowledgment of our work. We share their view that further multicenter prospective studies with standardized protocols are needed to refine global recommendations for prostate biopsy. Our experience supports the continued transition toward the transperineal approach as the safer and more sustainable standard, particularly in the context of rising antimicrobial resistance and evolving antibiotic stewardship principles.

Sincerely,

Oussama G. Nasrallah, MD

On behalf of all authors

Division of Urology, Department of Surgery American University of Beirut Medical Center Beirut, Lebanon
